# Selective Capture and Continuous Recovery of Sulfur-Containing Molecules from Flowing Wastewater Using a Capillary Ag_2_Mo_3_O_10_·1.8H_2_O/Carbon Fiber Membrane System

**DOI:** 10.3390/membranes16030084

**Published:** 2026-02-26

**Authors:** Lei-Yang Xue, Chu-Ya Luo, Han-Mei Xu, Jia-Xin Hua, Xue Zhang, Lian-Wen Zhu, Jun Wu

**Affiliations:** 1 School of Biology and Chemical Engineering, Jiaxing University, Jiaxing 314001, China; 2 College of Advanced Materials Engineering, Jiaxing Nanhu University, Jiaxing 314001, China

**Keywords:** Ag_2_Mo_3_O_10_·1.8H_2_O/carbon hybrid framework, capillary-driven separation, selective adsorption, continuous resource recovery

## Abstract

This work presents a novel, membrane-inspired hybrid framework composed of Ag_2_Mo_3_O_10_·1.8H_2_O nanowires grown in situ on carbon fiber cloth (CFC) for the continuous and selective recovery of high-value sulfur-containing molecules from organic wastewater. The framework forms an integrated hierarchical porous network rich in micro-/nano-channels, which facilitates efficient, capillary-driven water transport. Owing to its mesoporous texture and specific Ag–S coordination affinity, the material shows exceptional selectivity toward sulfur-containing dyes, enabling rapid adsorption (>94% removal of methylene blue within 10 min) and high specificity in mixed solutions. The hybrid also exhibits excellent reusability, maintaining high recovery efficiency over repeated adsorption–desorption cycles. When configured into a continuous-flow system, the framework operates without external pressure and achieves a water transport rate of 1875 mL·h^−1^·m^−2^. These findings underscore the potential of the Ag_2_Mo_3_O_10_·1.8H_2_O/CFC hybrid as an efficient, scalable, and sustainable platform for resource-oriented wastewater treatment.

## 1. Introduction

Water resources are essential for human life and societal development. However, aquatic ecosystems are increasingly threatened by persistent and structurally rooted challenges, with nutrient pollution (e.g., from nitrogen and phosphorus) being particularly prominent [1]. Moving beyond conventional purification, a resource-oriented approach to organic wastewater treatment holds greater long-term value, as it seeks to transform pollutants into recoverable materials [2]. This strategy, often termed wastewater resource recovery, typically involves directing industrial, agricultural, and domestic effluents through tailored systems—using physical, chemical, or biological processes—to meet standards for reuse [3]. For example, integrated methods combining membrane separation, adsorption, and Fenton oxidation have been developed for treating printing and dyeing wastewater [4].

Adsorption is widely employed for dye wastewater treatment due to its simplicity, operational flexibility, and cost-effectiveness, making it one of the most practical means of removing toxic contaminants [5]. While effective for low-concentration soluble dyes, conventional adsorbents often suffer from low capacity, poor selectivity, difficult regeneration, and limited resource-recovery potential. Membrane technology has advanced considerably owing to its high separation efficiency, economic feasibility [6], environmental compatibility, and potential for approaching “zero liquid discharge” [7,8]. Nevertheless, membrane processes are frequently limited by high operational and maintenance costs, significant energy consumption, and membrane fouling. Other chemical methods, such as photocatalytic oxidation, often face challenges related to inadequate purification performance and complex operational requirements [9,10]. From a practical standpoint, an ideal resource-oriented wastewater-treatment system should be efficient, economical, robust, and environmentally sustainable, while enabling: (1) resource utilization, (2) continuous operation, and (3) selective separation. Hence, developing novel materials and technologies for water purification and resource recovery remains a critical research objective [11].

In recent years, micro-/nanostructured layered oxide composites have attracted considerable interest for adsorption and membrane-separation applications due to their hierarchical architecture [12,13,14,15,16], ease of functionalization, well-defined transport channels, high specific surface area, fully accessible active sites, and excellent stability [17]. These properties suggest that integrating layered oxides with complementary components can yield materials with enhanced and tunable selective-adsorption functionality.

The crystal structure of Ag_2_Mo_3_O_10_·1.8H_2_O consists of a layered arrangement formed by zigzag chains of edge-sharing MoO_6_ octahedra, interconnected via interlayer Ag^+^ ions. Because of the strong affinity of silver for sulfur, these interlayer Ag^+^ sites can selectively capture sulfur-containing organic dyes through Ag–S coordination, enabling specific separation of mixed dyes without complex surface modification [18,19]. Inspired by membrane-separation principles, we directly grew Ag_2_Mo_3_O_10_·1.8H_2_O nanowires on a flexible, porous substrate to create a continuous, integrated separation platform. This design effectively combines the selective-adsorption capability of the layered oxide with the structural and processing advantages of membrane technology, allowing simultaneous molecular recognition and continuous fluid transport. Such an architecture not only improves separation efficiency but also aligns with the scalable, modular nature of modern membrane systems, offering a promising strategy for developing advanced functional membranes for targeted resource recovery.

In this work, we developed a continuous-flow resource-recovery platform inspired by membrane processes, based on a hybrid Ag_2_Mo_3_O_10_·1.8H_2_O/carbon fiber cloth (CFC) framework. An integrated porous network was constructed by uniformly growing Ag_2_Mo_3_O_10_·1.8H_2_O nanowires over a large-area CFC substrate. The design merges molecular-level selectivity—imparted by Ag–S coordination—with passive, capillary-driven fluid transport analogous to membrane permeation, thereby enabling energy-efficient continuous operation. The system exhibits rapid and specific adsorption of methylene blue from mixed-dye solutions, along with excellent reusability, maintaining ~97% recovery efficiency over multiple cycles. This work presents a membrane-inspired strategy for the selective, continuous, and sustainable recovery of high-value molecules from wastewater.

## 2. Materials and Methods

### 2.1. Materials

Carbon fiber cloth (CFC) was obtained from Yixing Xingtan Composite Material Co., Ltd (Yixing, China). The following reagents were used as received: silver nitrate (AgNO_3_, Sinopharm Chemical Reagent Co., Ltd., AR, ≥99.8%, Shanghai, China), ammonium molybdate tetrahydrate ((NH_4_)_6_Mo_7_O_24_·4H_2_O, Shandong Keyuan Biochemical Co., Ltd., AR, ≥99.0%, Heze, China), nitric acid (HNO_3_, Shanghai Lingfeng Chemical Reagent Co., Ltd., 65 wt%, Shanghai, China), methylene blue (MB, Sinopharm Chemical Reagent Co., Ltd., 98%, Shanghai, China), rhodamine B (RhB, Sinopharm Chemical Reagent Co., Ltd., 98%, Shanghai, China), ethanol (Sinopharm Chemical Reagent Co., Ltd., AR, ≥99.7%, Shanghai, China), and n-propanol (Shanghai Macklin Biochemical Technology Co., Ltd., AR, ≥99%, Shanghai, China). Dimethyl Sulfoxide (Shanghai Macklin Biochemical Technology Co., Ltd., AR, 78.13 (MW), Shanghai, China). Deionized water was used in all experiments.

### 2.2. Preparation of the Ag_2_Mo_3_O_10_·1.8H_2_O/CFC Hybrid Framework

A AgNO_3_ solution was prepared by dissolving 0.24 g of AgNO_3_ in 12 mL of deionized water. Separately, 0.465 g of (NH_4_)_6_Mo_7_O_24_·4H_2_O was dissolved in 18 mL of deionized water. The AgNO_3_ solution was poured into the molybdate solution, and the mixture was stirred under ultrasonication until homogeneous. The pH of the resulting precursor solution was adjusted to 2 using 1 M HNO_3_. A piece of CFC (4 cm × 20 cm) was rolled and placed into a Teflon-lined autoclave, followed by the addition of the precursor solution. The hydrothermal reaction was conducted at 110 °C for 1 h. To increase the loading of Ag_2_Mo_3_O_10_·1.8H_2_O, the hydrothermal growth step was repeated once. After cooling to room temperature, the obtained hybrid framework was thoroughly rinsed with deionized water until the effluent reached neutral pH and was finally dried under vacuum at 80 °C.

### 2.3. Characterization

The morphology and microstructure of the samples were examined using a Hitachi S-4800 field-emission scanning electron microscope (SEM, Tokyo, Japan). Transmission electron microscopy (TEM) and high-angle annular dark-field scanning TEM (HAADF-STEM) analyses were performed on an FEI Tecnai G20 microscope (Portland, OR, USA). For TEM sample preparation, Ag_2_Mo_3_O_10_·1.8H_2_O was dispersed in ethanol by ultrasonication, and a drop of the diluted suspension was deposited onto carbon-coated copper grids. X-ray diffraction (XRD) patterns were recorded on a Shimadzu XRD-7000 (Kyoto, Japan). Raman spectra were acquired using a HORIBA LabRAM HR Evolution spectrometer (Paris, France) with 532 nm laser excitation over the range of 50–4000 cm^−1^. Fourier transform infrared (FT-IR) spectra were obtained using an infrared spectrometer (PerkinElmer Spectrum two, Waltham, MA, USA). UV-vis absorption spectra were measured with a Shimadzu UV-2550 spectrophotometer (Kyoto, Japan).

### 2.4. Adsorption and Continuous-Flow Separation Tests

The adsorption performance of the as-synthesized Ag_2_Mo_3_O_10_·1.8H_2_O powder was first evaluated. The product obtained from the hydrothermal reaction was collected by suction filtration, rinsed thoroughly with deionized water, and dried in a vacuum oven at low temperature. After 24 h, the dried sample was ground into a powder using an agate mortar. Subsequently, 0.05 g of the powder was immersed in 30 mL of a methylene blue (MB) solution (10 mg·L^−1^) under stirring. Every 5 min, 3 mL of the supernatant was sampled, centrifuged, and its absorbance at 664 nm was measured using a UV-vis spectrophotometer to determine the residual MB concentration. The adsorption capacity at each time point was calculated accordingly.

The adsorption performance of the hybrid framework toward different dyes was evaluated. A piece of the Ag_2_Mo_3_O_10_·1.8H_2_O/CFC hybrid framework (5 cm × 3 cm) was immersed in 50 mL of each dye solution—methylene blue (MB), Rhodamine B (RhB), Evans Blue (EB), Methyl Orange (MO), and Acidic K (Ka)—at a concentration of 10 mg·L^−1^. The systems were kept in darkness for 12 h, after which the residual dye concentrations were analyzed by UV-vis spectrophotometry, and the adsorption isotherms were derived.

A capillary-driven continuous-flow separation system was constructed using a strip of the hybrid framework (4 cm × 20 cm). One end of the framework was immersed in a 200 mL beaker containing an MB/RhB mixed solution, while the other end was placed in contact with the inner wall of an empty 100 mL beaker. Driven by capillary action, the solution flowed continuously along the framework. The dye concentrations in the feed and collection beakers were periodically monitored by UV-vis spectrophotometry. The entire process was repeated in multiple independent experiments.

Finally, the Ag_2_Mo_3_O_10_·1.8H_2_O/CFC framework (4 cm × 20 cm) was soaked in 50 mL of dimethyl sulfoxide (DMSO) for 30 s, retrieved, and reused in the purification of flowing water over seven consecutive cycles. The recovery efficiency in each cycle was analyzed by UV-vis spectrophotometry.

### 2.5. Batch Adsorption Tests

The adsorption of methylene blue by Ag_2_Mo_3_O_10_·1.8H_2_O powder was investigated through isotherm and kinetic studies. Equilibrium data obtained under conditions of 10 mg·L^−1^ MB (23 mL) and 0.05 g adsorbent were modeled using the Langmuir equation. The adsorption kinetics were analyzed with a pseudo-first-order model. Subsequently, the effect of solution pH on the adsorption performance of Ag_2_Mo_3_O_10_·1.8H_2_O powder was examined. Simultaneously, the effect of non-thiol-positive dyes on the adsorption by Ag_2_Mo_3_O_10_·1.8H_2_O/CFC was tested using a UV spectrophotometer.

## 3. Results and Discussion

### Formation of the Ag_2_Mo_3_O_10_·1.8H_2_O/CFC Framework

CFC possesses a robust skeleton with high mechanical strength, excellent thermal stability, and notable corrosion resistance [20]. The aligned fiber bundles create well-defined linear nanochannels between adjacent fibers, forming an ordered network of nanofluidic pathways. Strong capillary forces within these channels, together with the intrinsic hydrophobicity of the carbon fiber matrix, provide an effective platform for generating and sustaining continuous water flow [21]. Experimentally, a CFC sample (4 cm × 20 cm) exhibited a water transport rate of up to 2188 mL·h^−1^·m^−2^, confirming its high fluid-conveyance efficiency. Owing to these structural and functional advantages, CFC was selected as the scaffold for constructing the Ag_2_Mo_3_O_10_·1.8H_2_O/carbon hybrid framework.

**Figure 1:**
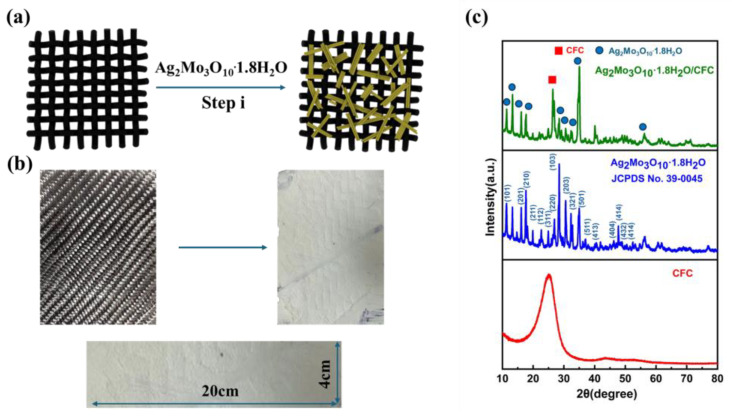
(**a**) Schematic of the Ag_2_Mo_3_O_10_·1.8H_2_O/CFC hybrid framework fabrication process. (**b**) Optical images of pristine CFC (**left**) and the resulting framework (**right**). (**c**) XRD patterns of the products.

As schematically illustrated in Figure 1a, the Ag_2_Mo_3_O_10_·1.8H_2_O/CFC hybrid framework was fabricated through a one-step hydrothermal process (step i, Figure 1a), during which Ag_2_Mo_3_O_10_·1.8H_2_O nanowires were directly grown on the CFC substrate. To increase the nanowire loading density, the hydrothermal treatment was performed twice—a critical step that enabled uniform, high-density, and large-area coverage of nanowires on the CFC support, thereby ensuring optimal functional performance of the resulting hybrid material. A representative Ag_2_Mo_3_O_10_·1.8H_2_O/carbon fiber hybrid framework sample, covering an area of 80 cm^2^, is shown in Figure 1b. The dimensions and geometry of this composite can be readily customized to meet specific application requirements, while the Ag_2_Mo_3_O_10_·1.8H_2_O phase exhibits the ability to nucleate and grow uniformly over large areas of the CFC substrate at high density. After two successive hydrothermal treatments, the mass loading of Ag_2_Mo_3_O_10_·1.8H_2_O reached 20 mg·cm^−2^, demonstrating its potential for scalable industrial implementation.

Structural analysis by XRD further confirms the formation of the hybrid framework (Figure 1c). Before nanowire growth, the diffraction peak at 25.2° corresponds to the characteristic (002) reflection of the carbon fiber substrate, consistent with previously reported data [22]. After the hydrothermal synthesis, a set of distinct new diffraction peaks appears at 11.4°, 13.3°, 16.1°, 17.7°, 28.5°, 31°, and 35°, alongside the persistent CFC signature. These additional peaks match well with the reference patterns for crystalline Ag_2_Mo_3_O_10_·1.8H_2_O [23,24,25], confirming the successful integration of Ag_2_Mo_3_O_10_·1.8H_2_O into the carbon fiber matrix and the establishment of a well-defined binary composite framework.

**Figure 2:**
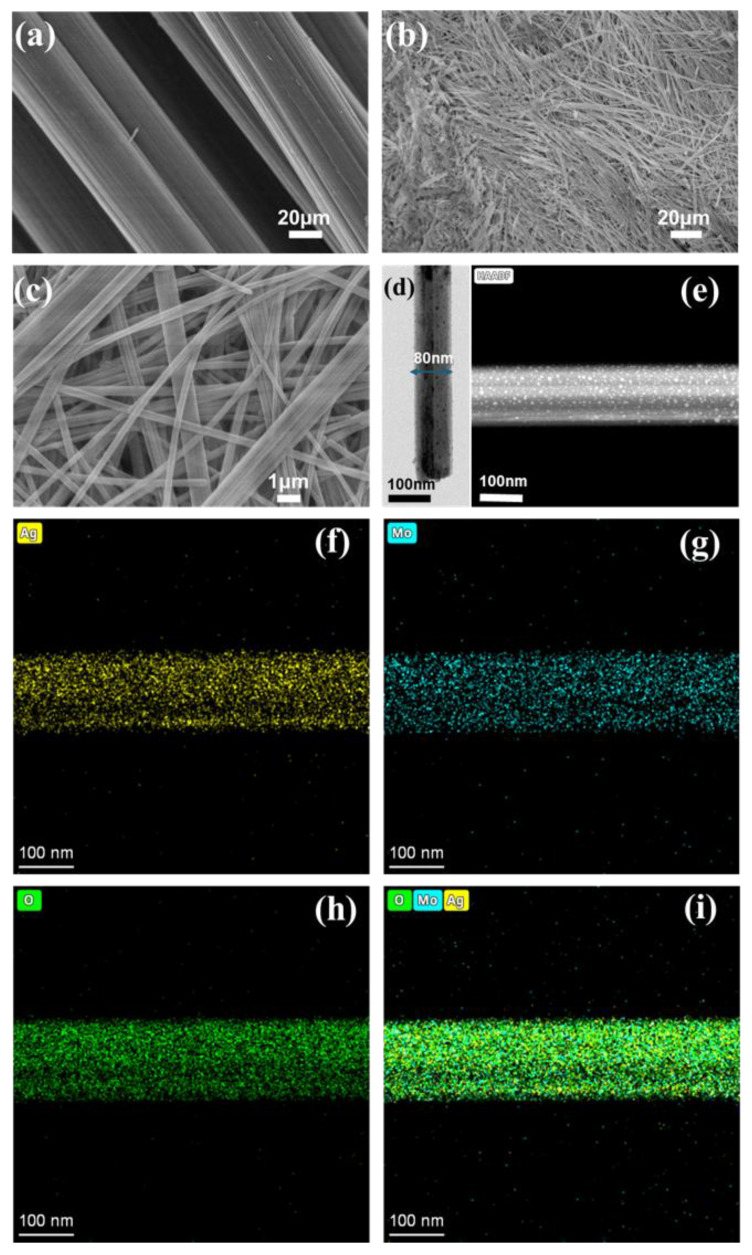
SEM images of (**a**) CFC and (**b**,**c**) Ag_2_Mo_3_O_10_·1.8H_2_O/CFC. (**d**) TEM image of an individual Ag_2_Mo_3_O_10_·1.8H_2_O nanowire. (**e**–**i**) HAADF-STEM images and element-sensitive mapping of the Ag_2_Mo_3_O_10_·1.8H_2_O nanowire.

The SEM image in Figure 2a shows the surface morphology of the CFC, revealing an interwoven network of ultra-long carbon fiber bundles. With an approximate thickness of 60 µm, these bundles serve as a robust and scalable scaffold for extensive Ag_2_Mo_3_O_10_·1.8H_2_O deposition. Figure 2b,c further display the surface morphology of the Ag_2_Mo_3_O_10_·1.8H_2_O/CFC composite, illustrating that the grown nanowires not only cover the outer surface of the CFC but also penetrate deeply into its interior, forming an interlocking network with the underlying carbon fibers [26,27]. As shown in the TEM image (Figure 2d), individual nanowires exhibit a uniform width of approximately 80 nm. These one-dimensional nanostructures interpenetrate and overlap, creating an integrated porous architecture with abundant interconnected micro- and nano-channels that function as effective pathways for capillary-driven fluid transport. Water transport measurements conducted on a 4 cm × 20 cm Ag_2_Mo_3_O_10_·1.8H_2_O/CFC sample revealed a transport rate of 1875 mL·h^−1^·m^−2^, which is 313 mL·h^−1^·m^−2^ lower than that of pristine CFC, likely due to partial pore occupation by the nanowire network.

To further confirm the composition and structure of the nanowires, HAADF-STEM imaging and elemental mapping were performed. As shown in Figure 2e–i, these techniques provide atomic-scale visualization of the elemental distribution, where the image contrast approximately scales with the square of the atomic number (Z^2^). Within the Ag_2_Mo_3_O_10_·1.8H_2_O nanowires, regions corresponding to silver (Z = 47) appear distinctly brighter than those of molybdenum (Z = 42) and oxygen (Z = 8), in agreement with the expected compositional variation [28]. Together with the XRD results, these observations confirm that the nanowires are composed of crystalline silver trimolybdate hydrate (Ag_2_Mo_3_O_10_·1.8H_2_O).

**Figure 3:**
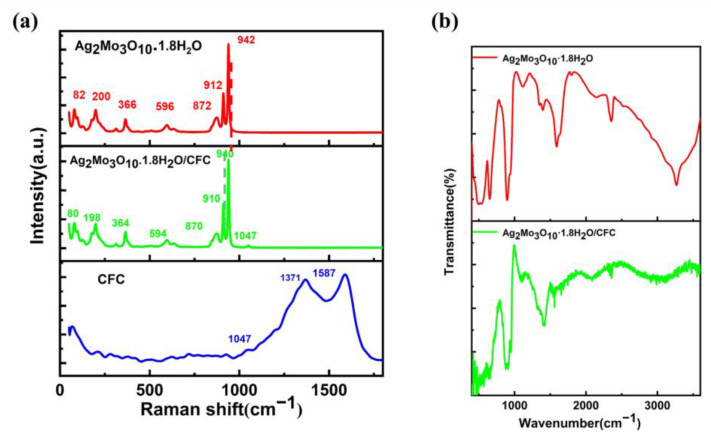
(**a**) Raman spectra of pristine carbon framework, Ag_2_Mo_3_O_10_·1.8H_2_O and Ag_2_Mo_3_O_10_·1.8H_2_O/carbon hybrid framework. (**b**) Diffuse reflectance spectra for Ag_2_Mo_3_O_10_·1.8H_2_O and Ag_2_Mo_3_O_10_·1.8H_2_O/carbon hybrid framework.

Figure 3a presents the corresponding Raman spectra. The peaks at 1371 and 1587 cm^−1^ correspond to the D and G bands of the carbon fibers, respectively. Characteristic peaks of Ag_2_Mo_3_O_10_·1.8H_2_O are observed at 940, 910, 870, 364, and 198 cm^−1^, which are assigned to the stretching vibrations of MoO_4_^2−^ units and the bending modes of O–Mo–O bonds [29]. The high intensity of these peaks indicates a well-crystallized and densely grown nanowire phase. In the Ag_2_Mo_3_O_10_·1.8H_2_O/CFC composite, subtle but consistent shifts in the Mo–O-related Raman peaks suggest an interfacial interaction between the two components. Complementary IR spectra (Figure 3b) further support this interaction. The O–H stretching band shifts from around 3300 cm^−1^ in pure Ag_2_Mo_3_O_10_·1.8H_2_O to approximately 3000 cm^−1^ in the composite. This shift, together with the retention of characteristic vibrational features between 500–1000 cm^−1^ [30], points toward structural integration, most likely mediated by hydrogen bonding between the surface hydroxyl/carboxyl groups on the carbon fiber cloth and the crystalline water in Ag_2_Mo_3_O_10_·1.8H_2_O. Collectively, the Raman and IR analyses confirm a distinct interfacial interaction, presumably through hydrogen bonding, which promotes structural coupling in the hybrid material.

**Figure 4:**
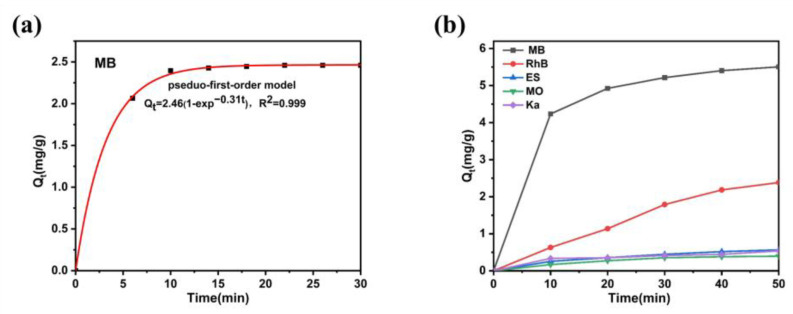
(**a**) Adsorption isotherm of pristine Ag_2_Mo_3_O_10_·1.8H_2_O adsorption of MB. (**b**) The adsorption of Ag_2_Mo_3_O_10_·1.8H_2_O/carbon hybrid framework of five different organic dyes.

The adsorption performance of Ag_2_Mo_3_O_10_·1.8H_2_O was evaluated using methylene blue (MB) as a model contaminant at a concentration of 10 mg/L (Figure 4a). Rapid initial uptake occurred on the external surfaces of the nanowires, followed by slower diffusion into their mesoporous interior. The adsorption kinetics were fitted to a pseudo-first-order model, with 94% MB removal achieved within 10 min, indicating efficient and rapid adsorption [31]. The curve is in good agreement with the Langmuir adsorption isotherm. In addition, we found that the optimal pH range for the adsorption of methylene blue by Ag_2_Mo_3_O_10_·1.8H_2_O was 7–8 (Figure S1). To assess selectivity, the 3 cm × 5 cm of Ag_2_Mo_3_O_10_·1.8H_2_O/CFC composite was tested against a series of dyes, including Methylene Blue (MB), Rhodamine B (RhB), Evans Blue (EB), Methyl Orange (MO), and Acidic K (Ka), each at 10 mg/L (Figure 4b). While strong and fast adsorption was observed for MB, only minimal uptake occurred for the other dyes. For instance, RhB exhibited only weak physical adsorption, likely attributable to the carbon fiber substrate itself. These results clearly demonstrate the high and selective adsorption capability of Ag_2_Mo_3_O_10_·1.8H_2_O toward MB [32]. The low removal efficiencies of Sudan III, Malachite Green and Methyl Red (Figure S2) clearly indicate that, despite their cationic nature and varied molecular sizes, none of these dyes were significantly captured under the same conditions where MB removal exceeded 94% within 10 min. Therefore, the control experiments support that the selective and efficient removal of MB is primarily governed by the specific Ag-S coordination interaction, rather than by general electrostatic attraction or size-exclusion effects.

**Figure 5:**
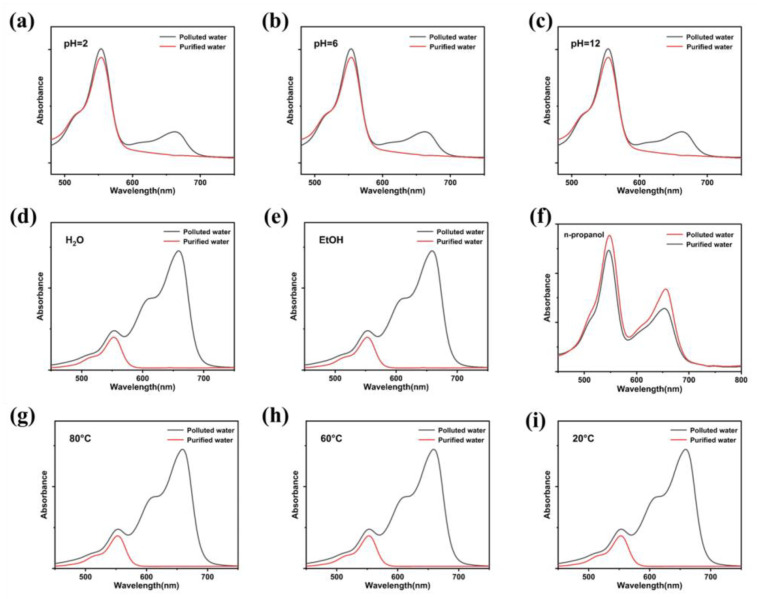
(**a**–**i**) UV-vis absorption spectra of Ag_2_Mo_3_O_10_·1.8H_2_O/CFC for flow water resource recovery testing of mixed dyes (MB and RhB) in different environments (pH, solvent, and temperature). (**i**) Curve of the recovery ratio of MB/RhB versus reuse times of Ag_2_Mo_3_O_10_·1.8H_2_O/CFC.

The hybrid framework was further tested in a continuous flow setup using mixed MB/RhB solutions. Under varying pH conditions (Figure 5a–c), the characteristic MB absorption peak at 663 nm was entirely absent in the filtrate, indicating near-quantitative removal (>99%). In contrast, only about 10% of RhB was retained, consistent with weak physical adsorption within the mesoporous network. The composite also exhibited high MB capture efficiency (>99%) in both aqueous and ethanolic MB/RhB mixtures (Figure 5d–f), whereas in n-propanol the removal efficiency decreased to 32%, likely due to competitive dehydrogenation reactions catalyzed by surface Ag sites. Moreover, the material maintained stable separation performance over a range of temperatures (Figure 5g–i), confirming its thermal robustness and consistent selectivity under different environmental conditions.

**Figure 6:**
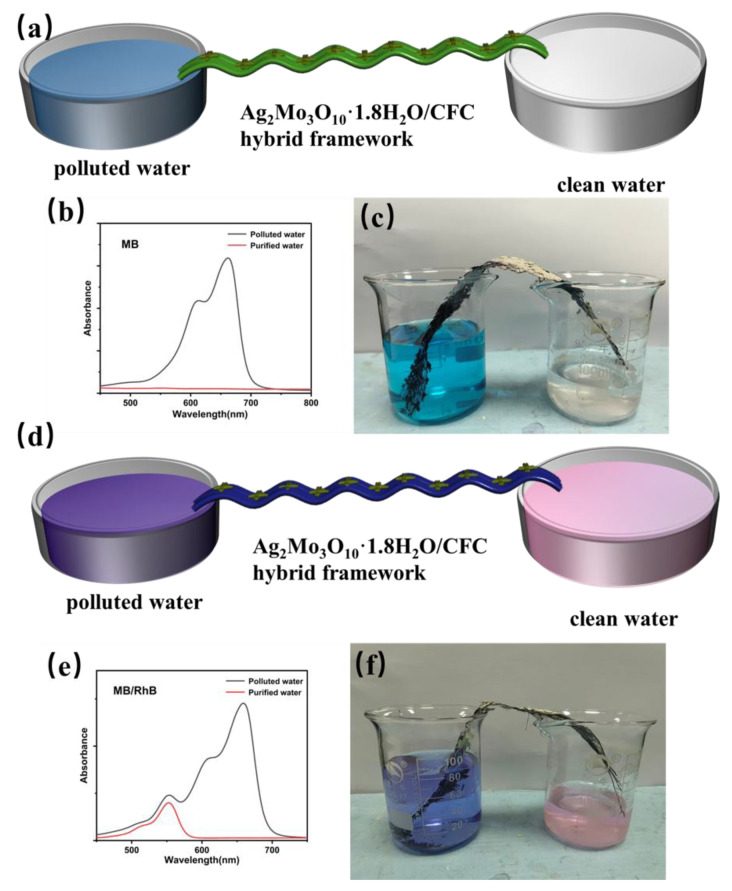
(**a**,**d**) Schematic diagram of the continuous flow water resource recovery system. (**b**,**e**) UV-vis absorption spectra of polluted water and purified water. (**c**,**f**) Digital photograph of the continuous flow water resource recovery system.

The findings above demonstrate that the Ag_2_Mo_3_O_10_·1.8H_2_O/CFC hybrid framework combines rapid selective adsorption, structural stability, and scalability, underscoring its strong potential as a functional material for water purification. Its inherent interconnected porous network and well-defined capillary channels provide efficient nanofluidic pathways for continuous, capillary-driven water transport. As illustrated in Figure 6a, a continuous-flow purification system was constructed based on this framework. One end of the material was immersed in a methylene blue (MB) solution, while the other was connected to an empty collection vessel. Driven solely by capillary action, the dye solution wicked upward through the framework, where MB molecules were rapidly captured by the Ag_2_Mo_3_O_10_·1.8H_2_O nanowires, allowing only clean water to reach the collector. This effective purification was confirmed visually and by UV-vis spectroscopy, which showed the complete disappearance of the characteristic MB peak in the collected water (Figure 6b,c). Notably, when fed with an MB/Rhodamine B (RhB) mixed solution, the system achieved selective molecular separation under capillary action alone, without external energy input. The collected solution exhibited a distinct red color (Figure 6f), and its UV-Vis spectrum (Figure 6e) revealed nearly complete retention of the RhB peak alongside the total absence of the MB signal, indicating efficient capture of MB and unobstructed passage of RhB. These results highlight the ability of the Ag_2_Mo_3_O_10_·1.8H_2_O/CFC hybrid to achieve continuous, selective separation of solutes in a flow-through configuration by leveraging its intrinsic capillary transport and specific affinity for MB. The core novelty of this work lies in the implementation of a selective Ag–S coordination mechanism within a capillary-driven, continuous-flow, multifunctional membrane system. Unlike previously reported static or pressure-driven Ag_2_Mo_3_O_10_-based membranes (Table S1), this architecture operates without external energy input, enables direct treatment of flowing wastewater, and achieves efficient contaminant removal under dynamic conditions, thereby bridging selective chemisorption with passive, high-throughput separation.

**Figure 7:**
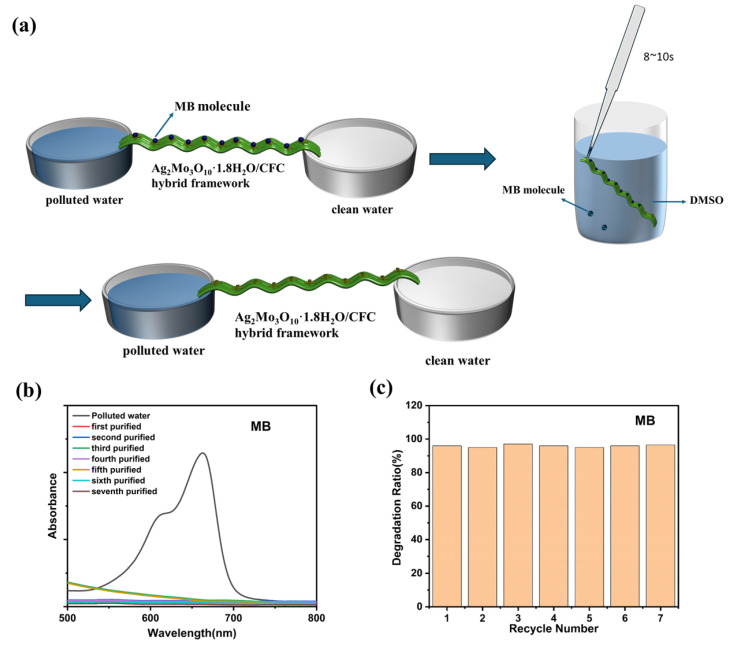
(**a**) Schematic diagram of material recycling and reuse. (**b**) UV-vis absorption spectra of MB solution over seven cycles. (**c**) Recovery efficiency across seven cycles.

Figure 7a–c demonstrate the reusability of the Ag_2_Mo_3_O_10_·1.8H_2_O/CFC hybrid framework, a critical feature for practical applications. The high selectivity for methylene blue (MB) stems from specific Ag–S coordination between silver sites on the nanowires and MB molecules [33]. Notably, this interaction is reversible under mild conditions. Control experiments using pristine carbon fiber cloth (CFC) alone showed minimal dye removal (5–15%), confirming that capillary action alone cannot explain the high MB selectivity. The efficient and selective removal in the Ag_2_Mo_3_O_10_·1.8H_2_O/CFC hybrid system is instead driven by specific Ag–S coordination (Figure S3). A 4cm × 20 cm hybrid framework was tested over multiple cycles. After each adsorption experiment, the material was regenerated by briefly immersing it in dimethyl sulfoxide (DMSO) for 30 s, allowing for rapid MB desorption (Figure S4). As shown in Figure 7b, the UV-Vis spectra of the purified water remained nearly identical over seven consecutive cycles, with the characteristic MB peak consistently absent. Accordingly, the calculated removal efficiency stayed above 97% throughout all cycles (Figure 7c). The Ag_2_Mo_3_O_10_·1.8H_2_O/CFC hybrid framework exhibited stable performance (~97% MB removal) and retained its crystallinity over seven consecutive adsorption-regeneration cycles in the capillary-driven continuous flow system (Figure S5). These results confirm that the Ag_2_Mo_3_O_10_·1.8H_2_O/CFC hybrid can be regenerated quickly and effectively without compromising its structural integrity or adsorption capacity. The combination of high selectivity, capillary-driven operation, and excellent recyclability highlights the material’s strong potential for sustainable, energy-efficient separation and recovery of sulfur-containing molecules in continuous-flow systems.

## 4. Conclusions

In summary, we have developed a novel Ag_2_Mo_3_O_10_·1.8H_2_O/carbon fiber cloth (CFC) hybrid framework by densely and uniformly growing Ag_2_Mo_3_O_10_·1.8H_2_O nanowires over a large-area CFC substrate. The resulting architecture—an interwoven network of nanowires and carbon fibers—forms a hierarchical porous structure rich in micro- and nano-channels, which functions as an efficient capillary-driven water transport platform with a flow rate of 1875 mL·h^−1^·m^−2^. Owing to specific Ag–S coordination, the hybrid framework exhibits rapid and selective adsorption toward sulfur-containing molecules, removing 94% of methylene blue (10 mg/L) within 10 min. The material also shows excellent selectivity in mixed-dye systems, along with outstanding reusability (~97% recovery over multiple cycles) and stability under varying environmental conditions. Furthermore, a continuous-flow resource recovery system based on this framework was demonstrated, enabling simultaneous water purification and targeted molecule recovery without external energy input. Together, these characteristics position the Ag_2_Mo_3_O_10_·1.8H_2_O/CFC hybrid as a highly promising and scalable material for advanced water resource recovery applications.

## Data Availability

The original contributions presented in this study are included in the article. Further inquiries can be directed to the corresponding authors.

## Supplementary Materials

The following supporting information can be downloaded at: https://www.mdpi.com/article/10.3390/membranes16030084/s1, Figure S1: (a) Adsorption isotherm of MB onto Ag_2_Mo_3_O_10_·1.8H_2_O powder: experimental data (points) fitted with the Langmuir model (line). (b) Effect of solution pH on MB removal efficiency using Ag_2_Mo_3_O_10_·1.8H_2_O powder as adsorbent; Figure S2: UV-Vis absorption spectra for the adsorption of non-sulfur cationic dyes on the Ag_2_Mo_3_O_10_·1.8H_2_O/CFC hybrid framework: (a) Sudan III, (b) Malachite Green (MG), and (c) Methyl Red (MR); Figure S3: The UV-Vis absorption spectra of the effluent collected during the continuous flow of (a) pure MB, (b) pure RhB, and (c) a mixed MB/RhB solution through pristine CFC; Figure S4: Regeneration and reusability of the Ag_2_Mo_3_O_10_·1.8H_2_O/CFC hybrid framework. (a) As-prepared hybrid framework before adsorption. (b) Framework after MB adsorption, immersed in dimethyl sulfoxide (DMSO) for desorption. (c) DMSO solution containing desorbed MB. (d) Regenerated hybrid framework ready for reuse, demonstrating retained structural integrity after the desorption cycle; Figure S5: Reusability and structural stability of the Ag_2_Mo_3_O_10_·1.8H_2_O film: (a–e) Digital photographs showing the film at different stages during seven consecutive adsorption–regeneration cycles; (f) UV-Vis absorption spectra of the methylene blue (MB) solution after each adsorption cycle; (i) XRD patterns of the film before use and after the seventh cycle, confirming the retention of crystalline structure; Table S1: Performance comparison of the Ag_2_Mo_3_O_10_·1.8H_2_O/CFC hybrid framework with representative membrane-based separation systems reported in the recent literature; Video S1: Membrane Regeneration and Reusability: A Four-Step Demonstration.
