# Supplementary material for: Selective Capture and Continuous Recovery of Sulfur-Containing Molecules from Flowing Wastewater Using a Capillary Ag2Mo3O10·1.8H2O/Carbon Fiber Membrane System
Source: Membranes (Basel). 2026 Feb 26;16(3):84. doi: 10.3390/membranes16030084 (PMC13028119; doi:10.3390/membranes16030084)
Supplement: Supplementary file 1 [file membranes-16-00084-s001.zip › membranes-4094601-supplementary.pdf]

Supporting Information

Selective Capture and Continuous Recovery of Sulfur-Containing Molecules from Flowing Wastewater Using a Capillary $Ag_2Mo_3O_{10}·1.8H_2O$/Carbon Fiber Membrane System

Lei-Yang Xue, Chu-Ya Luo, Han-Mei Xu, Jia-Xin Hua, Xue Zhang, Lian-Wen Zhu and Jun Wu

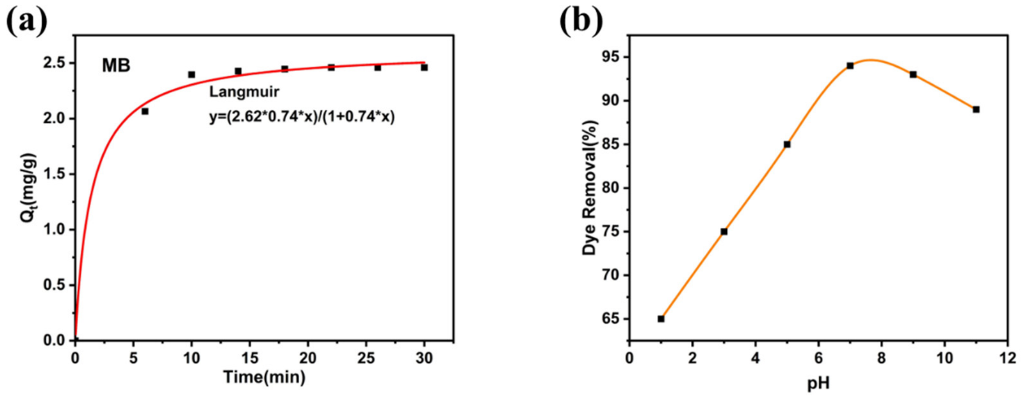

Figure S1. (a) Adsorption isotherm of MB onto $Ag_2Mo_3O_{10}·1.8H_2O$ powder: experimental data (points) fitted with the Langmuir model (line). (b) Effect of solution pH on MB removal efficiency using $Ag_2Mo_3O_{10}·1.8H_2O$ powder as adsorbent.

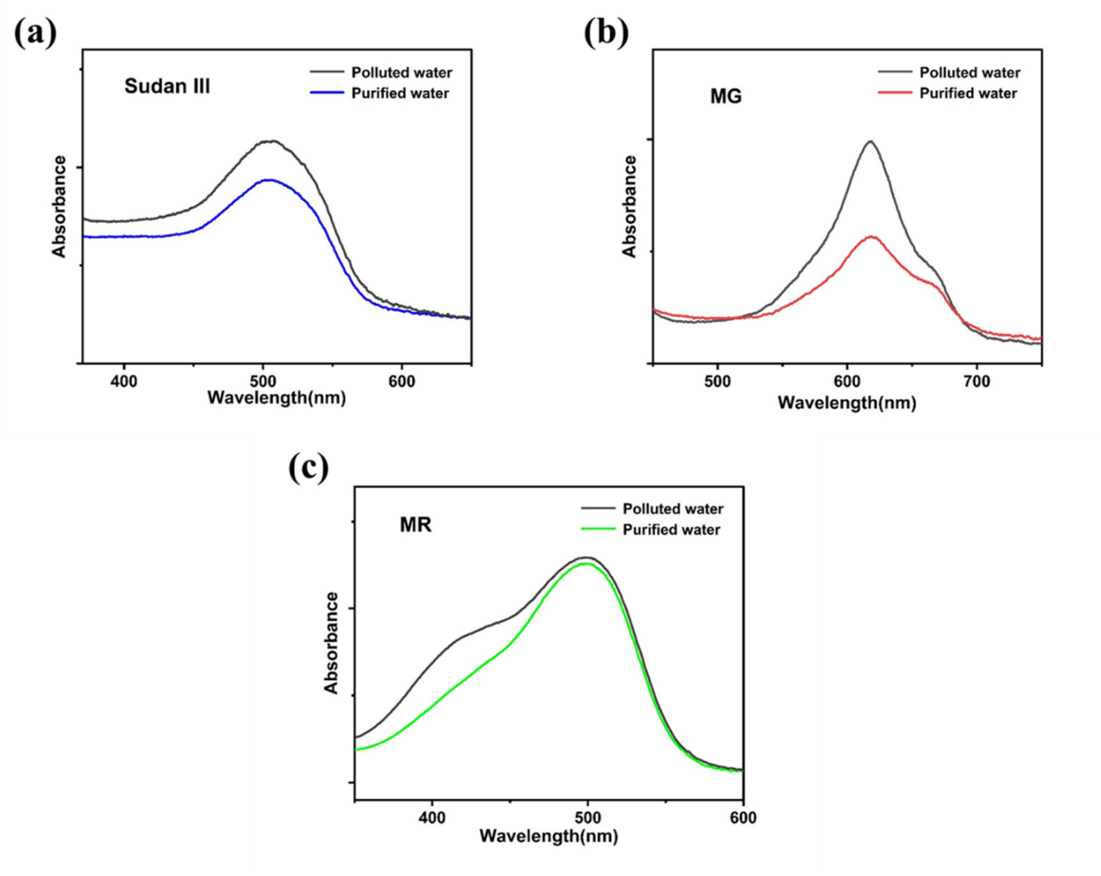

Figure S2. UV-Vis absorption spectra for the adsorption of non-sulfur cationic dyes on the $Ag_2Mo_3O_{10}\cdot 1.8H_2O$/CFC hybrid framework: (a) Sudan III, (b) Malachite Green (MG), and (c) Methyl Red (MR).

Table S1. Performance comparison of the $Ag_2Mo_3O_{10}·1.8H_2O$/CFC hybrid framework with representative membrane-based separation systems reported in the recent literature.

Literature	Category	Novelty1	Novelty2
Nanoscale, 2023, 15, 6607.	Membrane separation	Static water<br>23.1 L $m^{-2}·h^{-1}·bar^{-1}$<br>Separation efficiency: 99.93%	Energy consumption (<4 kPa)
Chemical Engineering Journal,<br>2026, 17, 3455.	Membrane separation	Static water<br>75 L $m^{-2}·h^{-1}·bar^{-1}$<br>Separation efficiency: 90%	Energy consumption 200kpa
Chemical Engineering Journal,<br>2026, 10, 1016.	Membrane separation	Static water<br>1201.4 L $m^{-2}·h^{-1}·bar^{-1}$<br>Separation efficiency: 99.85%	Energy consumption 90kpa
Chemical Engineering Journal, 2026, 10, 1039.	Membrane separation	Static water<br>443.7 L $m^{-2}·h^{-1}·bar^{-1}$<br>Separation efficiency: >98 %	Energy consumption 80kpa
This work	Multifunctional membrane system	Flowing water<br>1875 $L·h^{-1}·m^{-2}$<br>Separation efficiency: 97%	No energy consumption

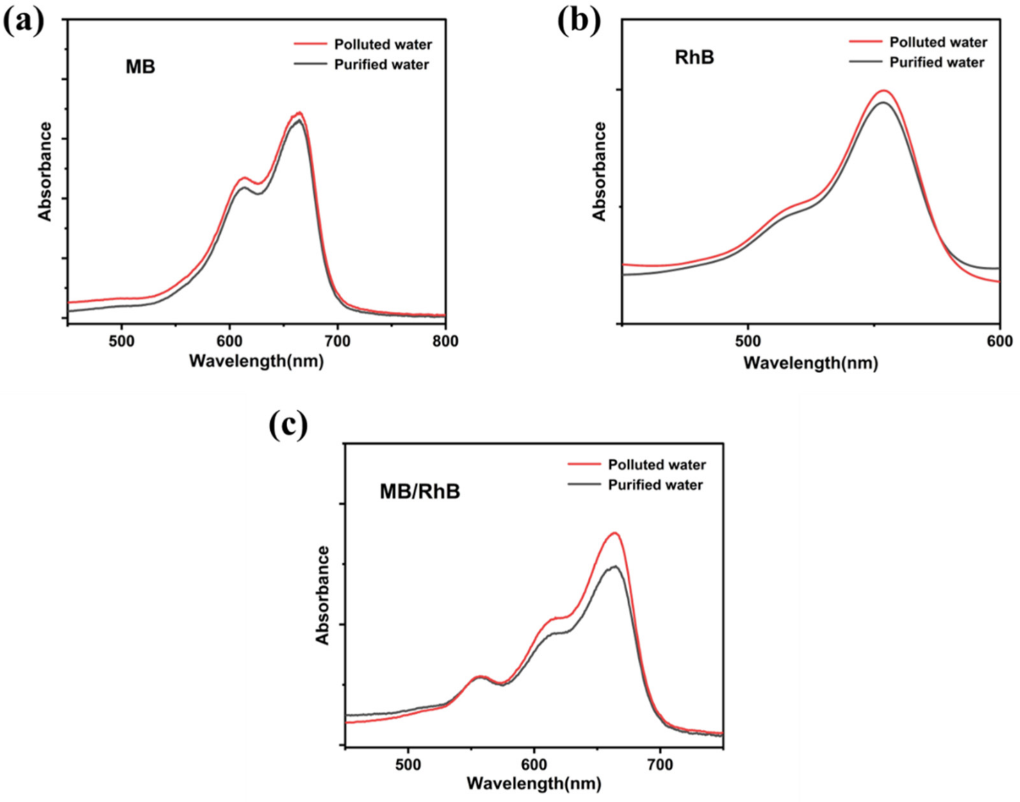

Figure S3. The UV-Vis absorption spectra of the effluent collected during the continuous flow of (a) pure MB, (b) pure RhB, and (c) a mixed MB/RhB solution through pristine CFC.

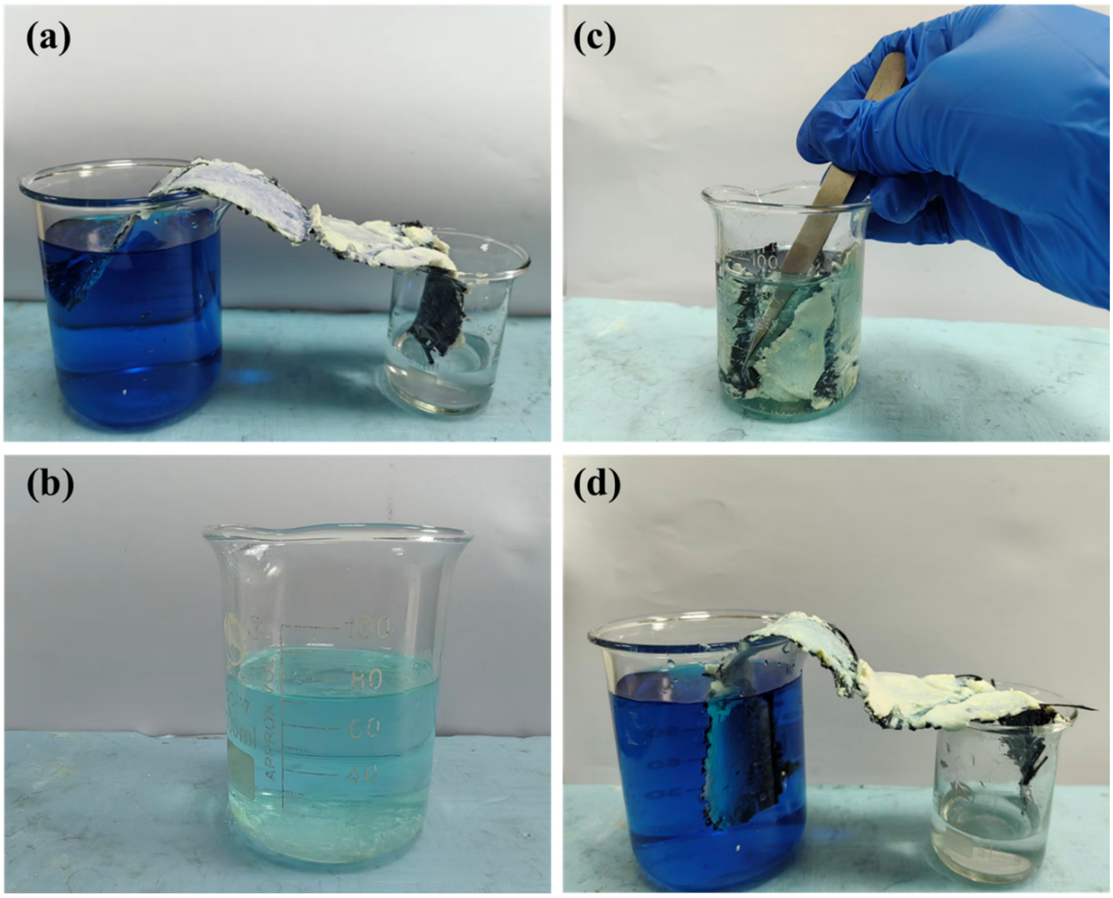

Figure S4. Regeneration and reusability of the $Ag_2Mo_3O_{10}\cdot 1.8H_2O$/CFC hybrid framework. (a) As-prepared hybrid framework before adsorption. (b) Framework after MB adsorption, immersed in dimethyl sulfoxide (DMSO) for desorption. (c) DMSO solution containing desorbed MB. (d) Regenerated hybrid framework ready for reuse, demonstrating retained structural integrity after the desorption cycle.

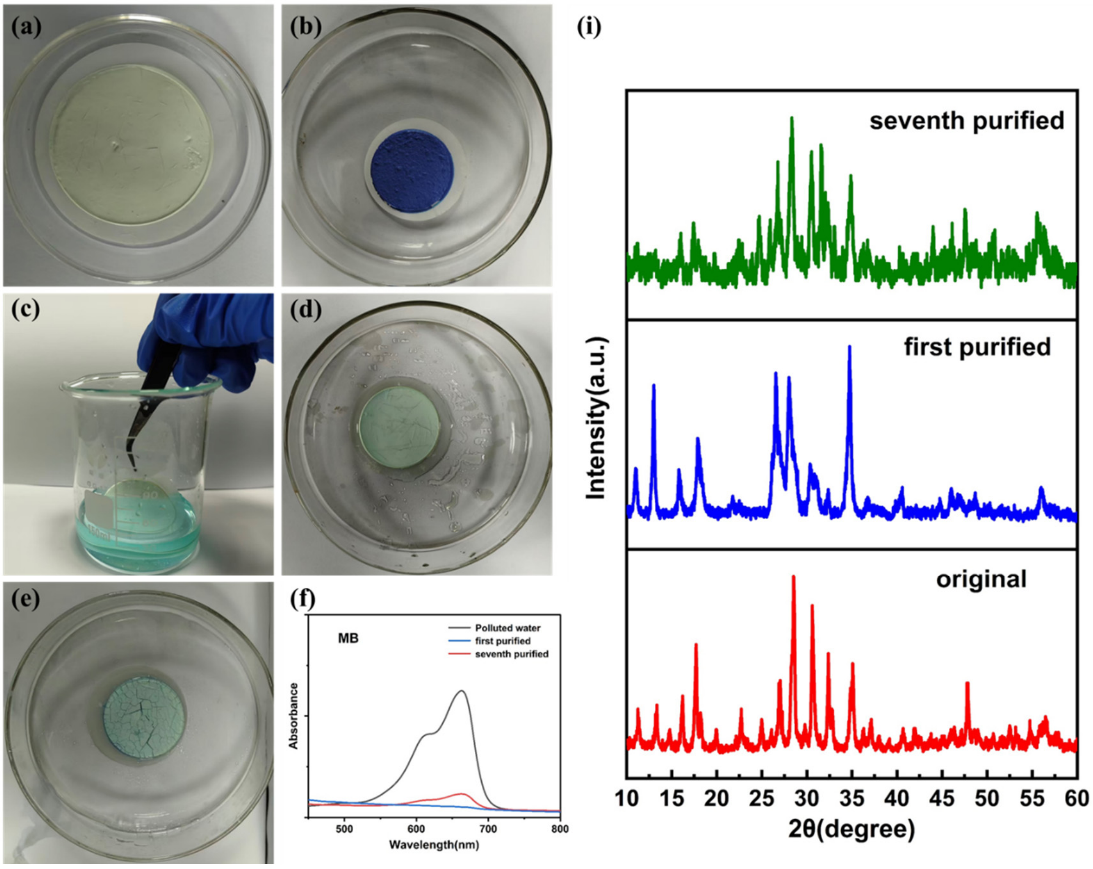

Figure S5. Reusability and structural stability of the $Ag_2Mo_3O_{10}·1.8H_2O$ film: (a–e) Digital photographs showing the film at different stages during seven consecutive adsorption–regeneration cycles; (f) UV-Vis absorption spectra of the methylene blue (MB) solution after each adsorption cycle; (i) XRD patterns of the film before use and after the seventh cycle, confirming the retention of crystalline structure.
